# Sickle Cell Disease in Africa: A Neglected Cause of Early Childhood Mortality

**DOI:** 10.1016/j.amepre.2011.09.013

**Published:** 2011-12

**Authors:** Scott D. Grosse, Isaac Odame, Hani K. Atrash, Djesika D. Amendah, Frédéric B. Piel, Thomas N. Williams

**Affiliations:** a Division of Blood Disorders, National Center on Birth Defects and Developmental Disabilities, CDC, Atlanta, Georgia; b Division of Haematology/Oncology, Hospital for Sick Children, the Department of Pediatrics, Faculty of Medicine, University of Toronto, Toronto, Canada; c African Population and Health Research Center, Nairobi, Kenya; d KEMRI Centre for Geographic Medicine Research, Kilifi, Kenya; e INDEPTH Network of Demographic Surveillance Sites, Accra, Ghana; f Spatial Ecology and Epidemiology Group, the Department of Zoology, University of Oxford, Oxford, United Kingdom; g Department of Pediatrics and Nuffield Department of Medicine, University of Oxford, Oxford, United Kingdom

## Abstract

Sickle cell disease (SCD) is common throughout much of sub-Saharan Africa, affecting up to 3% of births in some parts of the continent. Nevertheless, it remains a low priority for many health ministries. The most common form of SCD is caused by homozygosity for the β-globin S gene mutation (SS disease). It is widely believed that this condition is associated with very high child mortality, but reliable contemporary data are lacking. We have reviewed available African data on mortality associated with SS disease from published and unpublished sources, with an emphasis on two types of studies: cross-sectional population surveys and cohort studies. We have concluded that, although current data are inadequate to support definitive statements, they are consistent with an early-life mortality of 50%–90% among children born in Africa with SS disease. Inclusion of SCD interventions in child survival policies and programs in Africa could benefit from more precise estimates of numbers of deaths among children with SCD. A simple, representative, and affordable approach to estimate SCD child mortality is to test blood specimens already collected through large population surveys targeting conditions such as HIV, malaria, and malnutrition, and covering children of varying ages. Thus, although there is enough evidence to justify investments in screening, prophylaxis, and treatment for African children with SCD, better data are needed to estimate the numbers of child deaths preventable by such interventions and their cost effectiveness.

## Introduction

In Africa, sickle cell disease (SCD) is reported to be associated with a very high rate of childhood mortality, 50%–90%, yet there is a lack of reliable, up-to-date information.^1–9^ The most frequently cited study was conducted in the Garki district in rural northern Nigeria in the early 1970s.^10–12^ This Garki study provided valuable information on child survival and other aspects of the natural history of SCD during early childhood for that area about 40 years ago, but current, up-to-date information on the burden of mortality from SCD in Africa among populations with access to currently available treatments and preventive interventions is lacking.

Quantifying the number of under-5 child deaths from SCD in African countries is important to attract policy support and resources for measures to reduce the burden of mortality. Elevated mortality due to SCD among children younger than 5 years has been virtually eliminated from North America through a number of simple interventions, including newborn screening and prevention of infection through the provision of penicillin prophylaxis and polyvalent pneumococcal vaccination.^13,14^ Even in the absence of these therapies, health education (e.g., raising awareness of fever and of splenic sequestration) has been demonstrated to be effective in reducing SCD mortality in young children.^15–17^ Moreover, a pilot program in Benin has shown that with newborn screening and good follow-up care, it is likewise possible to reduce mortality among African children with SCD to the same level as other children.^18^ One of the major challenges in evaluating the cost effectiveness of newborn screening for SCD is the lack of adequate data on the numbers of deaths in unscreened cohorts.^19,20^

The 63rd session of the UN General Assembly in December 2008 adopted a resolution on the “recognition of sickle-cell anaemia as a public health problem,” and urged Member States and UN organizations to raise awareness of SCD on June 19 of each year.^21^ Despite the fact that the UN has called for global efforts “to bring the disease out of the shadows,” relatively little attention has been given to assessing the burden of SCD and how to reduce it in Africa, where about 85% of children with SCD are born.^22^ In 2010, the WHO Regional Office for Africa proposed a SCD strategy in official recognition of the fact that this disease is an important cause of child mortality in many African countries.^23^ Enough is already known to justify investment in public health SCD programs, such as those already in place for other conditions (e.g., HIV or malaria), for promoting widespread screening, health education, and treatment to prevent or manage SCD complications.

## The Classification of Sickle Cell Disease

**Figure 1:**
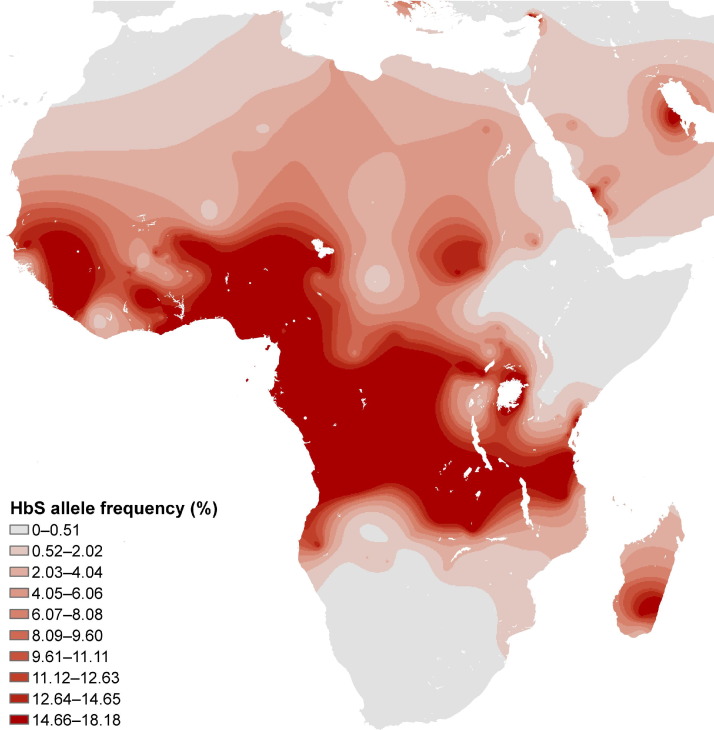
Map of the distribution of the β^S^ gene in Africa *Note:* The map is based on representative indigenous population samples and is adapted from Figure 1b in Piel et al.^24^ The figure shows the global distribution of the sickle cell gene and geographic confirmation of the malaria hypothesis.

The most common subtype of SCD worldwide is homozygous SCD, characterized by the presence of two copies of the β-globin S (β^S^) mutation that codes for sickle cell hemoglobin (Hb S). Homozygous SCD is variably referred to as sickle cell anemia, Hb SS, SS, SS disease, or sickle cell disease-SS. The distribution of the β^S^ allele (Figure 1) has recently been mapped globally using detailed geo-referenced data and displays a close association with the historical distribution of *Plasmodium falciparum* malarial endemicity.^24^ Within Africa, the frequency of β^S^, and accordingly SS, is highest in low-altitude equatorial regions. The second subtype of SCD common in Africa is compound heterozygosity for β^S^ and β^C^ (SC). The β^C^ allele is found almost exclusively among people of West African ancestry, being most common among those in Burkina Faso and northern Ghana. Compound heterozygosity with β^+^-thalassemia (Sβ^+^-thalassemia) is a form of SCD that is believed to be rare in most of sub-Saharan Africa.^12^ In Central, East, and Southern Africa, SCD is generally assumed to be synonymous with SS disease, although few studies have specifically looked for Sβ^0^-thalassemia.

## Mortality and Sickle Cell Disease in Africa

Reports of high rates of childhood mortality, 50%–90%, among African children with SCD are specific to SS.^1–7,10–12^ Qualitatively, experts have stated that the “majority,”^25^ “most,”^20,26^ the “vast majority,”^27^ or “nearly all”^28^ Africans born with SS die during childhood. From the 1950s onward, multiple researchers have reported an almost total absence of SS among samples of African adults,^10,29–33^ whereas other investigators have reported finding a frequency of SS among African adults of reproductive age of up to 20% of the expected number.^34–38^ The impact of SC on child survival is less clear. Evidence of raised mortality has been reported in some studies,^39^ but not in others.^40^

The purposes of this review are: (1) to describe the methods that can be used to estimate SCD mortality; (2) to assess the available data on child survival among SS individuals in Africa; and (3) to discuss prospects for improving the current estimates through the collection and analysis of additional data. We reviewed and interpreted recent data that could provide insights into the current rates of survival among children with SCD in Africa, much of which has not been cited in previous expert discussions.

## Approaches to Estimating Mortality

Two basic epidemiologic approaches can be used to assess mortality among children with SCD or any other congenital condition. The first is a prospective cohort design in which a representative sample of infants are recruited in early life and are then followed to assess their subsequent risk of death. Such studies “are expensive and time-consuming”^41^ and, therefore, are relatively uncommon. The second approach to estimating mortality among patients with SCD is to collect cross-sectional data on children of various ages and compare the frequencies of hemoglobin genotypes across age groups. To the extent that children with SS experience excess mortality relative to children with other genotypes, the frequency of that genotype will decrease progressively with age.^41^

Because the number of infants included in cross-sectional surveys typically is too small for precise estimates of the frequency of SS, an alternative is to use estimates of the frequency of the β^S^ allele among the population, together with the Hardy-Weinberg equilibrium (HWE) formula, to estimate the frequency of SS at birth.^22,38^ This method assumes either random mating or the availability of data on the population coefficient of consanguinity. It also assumes that data are available on heterozygosity frequencies at birth, or that the heterozygous state does not markedly influence overall survival. Although the sickle cell trait or carrier state (AS) can reduce the risk of severe *P. falciparum* malaria by roughly 90%^42^ and is associated with reduced all-cause mortality at aged 2–16 months,^43^ the prevalence of AS does not appear to be significantly affected as a result of the heterozygote advantage.^11^ Leaving aside those complications, the HWE formula in regard to β^S^ and β^C^, the major alleles that contribute to SCD, is represented by Equation 1:

(1) $${\text{p}}^{2}+2\text{p}\text{q}+{\text{q}}^{2}+2\text{p}\text{r}+{\text{r}}^{2}+2\text{q}\text{r}=1$$

(1)

for 2 and 3 alleles, where p=β^A^ allele frequency; q=β^S^ allele frequency; r=β^C^ allele frequency, and 2pq, 2pr, and 2qr are the frequencies of AS, AC, and SC, respectively.

If there are only two alleles present, it is possible to calculate the expected frequency of SS (q^2^) from the observed frequency of AS (2pq=n) using Equation 2:

(2) $$\text{q}=\frac{1-\sqrt{1-\text{Z}\text{n}}}{\text{Z}}.$$.

(2)

For example, among a population in which the observed prevalence of AS (n) is 20.0%, and no other hemoglobin variants are present, on the basis of HWE, it is estimated that the β^S^ allele frequency (q) is 0.113, and the expected frequency of SS (q^2^) is 1.27% (Table 1).

## Prospective Cohort Studies

A major limitation of prospective cohort studies for assessing the population probability of survival is that if medical follow-up is offered for children diagnosed with SCD, survival is likely to be much higher than that experienced by children with SCD in the population.^41^ This is likely to occur even if children are not prospectively screened for SCD, because of the close medical attention received by all children in prospective clinical studies.

Five prospective cohort studies involving infants in Africa with SCD were identified. Two older studies did not report systematic follow-up nor complete data on survival.^11,42^ Two recent studies with systematic follow-up, one each from Kenya^43^ and Ghana,^44^ both reported little or no excess mortality among children with SS relative to children with other genotypes. First, the Asembo Bay Cohort Project in Kisumu, Kenya,^43^ during 1992–1994 retrospectively genotyped specimens and reported mortality for children with hemoglobin genotypes AA, AS, and SS. A total of 1022 children were followed prospectively, of whom 1002 were genotyped. Overall, the cumulative probability of survival to age 6 years adjusted for maternal survival and gestational age was 75% among children with SS, compared with 78% among children with AA and 81% among children with AS. Second, Kreuels et al.^44^ prospectively recruited 1070 Ghanaian infants aged 2–4 months during 2003 and followed 852 of them to age 23 months. A total of 19 children died, none of them with either SS or SC.

In urban Guinea-Bissau, infants at age 6 months were monitored for a prospective cohort study, and at age 18 months, 1057 of them were genotyped for hemoglobin variants.^45^ The authors identified 50 (4.7%) heterozygotes and 2 (0.2%) homozygotes, consistent with an expected SS frequency of 0.1% based on HWE. The children subsequently were followed through age 4 years, and neither child with SS died.

Other prospective cohort studies have enrolled patients of various ages at SCD clinics, an approach that does not provide information on survival from birth. For example, Makani et al.^8^ reported data on 169 children with SS younger than age 2 years attending a large SCD clinic in Dar-es-Salaam, Tanzania, among whom 2 (1.0%) deaths were recorded. The low rate of mortality was consistent with the observations of Aidoo et al.^43^ and Kreuels et al.^44^ of low mortality among young children with SS who are enrolled in high-quality prospective research studies.

## Age-Stratified Cross-Sectional Surveys

**Figure 2:**
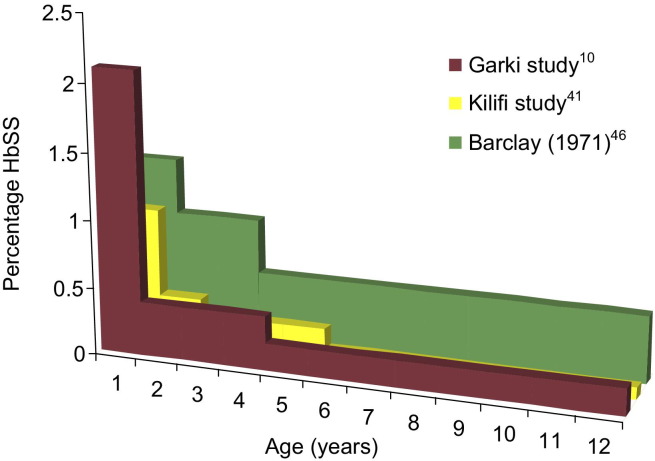
Prevalence of Hb SS by age among children in three African studies *Note:* The studies are Fleming et al.^10^ (Garki, Nigeria), Williams et al.^41^ (Kilifi, Kenya), and Barclay^46^ (Zambia). Hb SS, sickle cell anemia

The use of cross-sectional survey data to assess the burden of SCD mortality is not new,^38^ although only a handful of African studies have compared prevalence rates across age groups (Figure 2; Table 2). For example, Barclay^46^ reported that among 7569 children in a Zambian mining town, the frequency of SS decreased from 1.3% among 2845 infants to 0.9% among 2200 children aged 1–3 years, and to 0.5% among 2306 children aged 3–12 years, an apparent excess mortality of 60% relative to children with other genotypes (AA and AS together), calculated as (1.3−0.5)/1.3.

The Garki study that, as previously discussed, is cited widely in the context of discussions on SCD mortality,^12^ examined 534 newborns and 896 other children aged 1–14 years in a rural part of Kano State, Nigeria, in the early 1970s. The authors reported that the frequency of SS was 2.1% among newborns (11/534); 0.4% (1/259) at age 1–4 years; and 0.2% (1/637) at age 5–14 years.^10,11^ Those figures indicated a 92% cumulative excess mortality rate through age 15 years for those with SS relative to those with AA or AS. In a subsequent review, Fleming stated that at “5 years and above less than 2% of the expected numbers of subjects with Hb SS” were alive.^12^ However, that does not mean that 98% of subjects with SS disease died before the age of 5, as is often stated, because adults as well as children were included in the denominator. The probability of death before age 5 years can be calculated only through estimation of survival curves showing the percentage alive at each exact age, and that requires larger numbers of observations.

Recently published survey data from Kilifi district in coastal Kenya also reported a low frequency of SS in age groups beyond infancy.^25,41^ Among 1480 healthy control participants younger than age 5 years, McAuley et al.^25^ found a frequency of 1.0% SS (8 of 782) among infants aged 0–11 months and 0.3% (2 of 697) among children aged 12–59 months. In combination with data from the same cohort reported by Williams et al.,^41^ three (0.1%) of 3261 children aged 6–13 years had SS. The frequency of AS among children aged 0–59 months was 14.7% (218 of 1480),^25^ and according to HWE, the predicted frequency of SS was 0.64%. Therefore, the proportionate deficit among those in the group aged 6–13-years was approximately 85%.

Danquah et al.^47^ recently reported that among 2108 children younger than age 10 years in the Northern region of Ghana, the frequencies of AS and SS were 7.4% and 0.3%, respectively, consistent with HWE. The age-specific findings, which were shared in a personal communication (FP Mockenhaupt, Institute for Tropical Medicine, Berlin, personal communication, February 9, 2011), indicated that the frequency of SS was 0.39% among 1266 children younger than age 5 years and 0.12% among 842 children aged 5–9 years. These data suggested, therefore, an approximate 70% deficit of SS among children older than age 5 years. However, because of the low frequency of the β^S^ allele in northern Ghana, the study was too small to yield reliable estimates of age differences in prevalence.

## Other Cross-Sectional Surveys

Other cross-sectional surveys of hemoglobin variants among African populations have compared the observed prevalence of SS relative to that which would be expected based on the frequency of the β^S^ allele and HWE. In Burkina Faso, Simpore et al.^40^ found 12 (0.13%) homozygotes among 9201 individuals screened during 1997–1999, consistent with a deficit in the numbers of people with SS of approximately 50% relative to the number expected based on HWE with frequencies of AS and SC of 8.1% and 1.5%, respectively. In western Kenya, Desai and colleagues^48^ genotyped almost 1900 children younger than age 36 months during 1998–1999 and found frequencies of AS and SS of 22.0% and 0.6%, respectively. The expected frequency of SS was 1.6%, which suggest that approximately 60% of the children born with SS in the communities surveyed by Desai et al. had died prior to the health surveys. Finally, four small cross-sectional studies from rural populations in The Gambia and Senegal are also summarized in Table 2. The pooled prevalence of SS in the four studies^49–52^ was approximately 75% lower than the expected frequency of homozygotes based on HWE. It should be noted that these studies were conducted in isolated rural communities, and it is not possible to draw conclusions about survival probabilities in a country in the absence of national-level survey data.

## Discussion

To sum up, most studies that have included data about child survival among unselected or nonclinical samples of African children have suggested that the majority of those with SS died during early childhood, as indicated by the substantial deficit of SS among samples of older children or adults. However, most published information comes from rural areas within a handful of countries, as exemplified by the Garki study conducted in northern Nigeria in the early 1970s.^10,11^

Although such studies provide vitally important information on the natural history of SCD in these selected areas, they do not shed light on how the burden of SCD mortality might have changed over time as medical care and public health have improved. There are some data to suggest that survival might be improving. For example, in a study conducted in Lusaka, Zambia, Athale and Chintu reported that the case-fatality rate among children with SS who were admitted to the University Teaching Hospital decreased from 18.6% in 1970 to 6.6% during 1987–1989.^53^ Nevertheless, it is very likely that the majority of children with SS in rural Africa still die during childhood according to contemporary evidence from recent cross-sectional studies, including research papers from central Burkina Faso by Simpore et al.,^40^ the Nyanza province of Kenya by Desai et al.,^48^ the Kilifi district in coastal Kenya by Williams et al.,^41^ and from the Northern region of Ghana by Danquah et al.^47^ Although prospective cohort studies by Aidoo et al.^43^ and Kreuels et al.^44^ from western Kenya and southern Ghana, respectively, indicated little excess mortality among homozygotes, such data may not generalizable, given the likelihood that enrollment into prospective cohort studies will raise the probability of survival.

Applied epidemiologic research on SCD and child survival among a variety of African populations is needed. Prospective intervention studies aimed at preventing morbidity and mortality associated with SCD within the context of African primary healthcare systems are urgently needed. Such studies should carefully document and evaluate the specific services provided and the cost of delivering such services in order to evaluate the scalability of interventions of proven efficacy. Although prospective cohort studies are useful for studying morbidity, they are costly to undertake and limited in scope and generalizability, making it unlikely that many will be conducted.^41^ More critically, because the clinical facilities and care available to children in such studies are much better than the medical services received by the general pediatric population, the survival rates observed among children enrolled in such studies are also likely to be much higher. Close medical supervision is itself an intervention.

The alternative to cohort studies is cross-sectional observational studies. Such studies have the advantage of studying the actual situation of child survival under prevailing conditions. However, in an age of proven life-saving value of antimicrobial prophylaxis, children with SCD discovered or recruited by organized research studies in Africa should be provided such therapy. It is not ethically acceptable to watch such children get infected and die from preventable causes without intervening. The same constraint does not apply to the re-use of specimens or data collected from studies fielded for other purposes. Creative approaches to the analysis of cross-sectional data from existing, large, representative surveys are needed.

Better data on the birth frequencies of SS and SC in Africa are needed. Frequencies of β^S^ alleles can vary considerably over relatively small distances (Figure 1).^24^ For example, pilot newborn screening studies in the Democratic Republic of the Congo have indicated that the frequency of SS at birth varies by region from 0.8% to 1.7%,^54^ and in neighboring Rwanda and Burundi, the frequency is considerably lower, 0.1% or less.^55,56^ Nevertheless, too few surveys have been conducted to allow for the estimation of birth frequencies for the whole of Africa.^26^ Large numbers of newborns need to be screened to estimate these frequencies with precision. An example of such a large-scale newborn screening has been conducted in Kumasi, Ghana, since 1993;^57^ its expansion to the rest of the country is expected to result in a large life-saving benefit.^20^

## Conclusion

Sickle cell disease is an important but largely neglected risk to child survival in most African countries. Action is needed on at least two fronts. First, and most important, public health programs including newborn screening, health education, and immunization are urgently needed on the model of pilot programs already being conducted in Benin and Ghana.^21,57^ Careful collection and analysis of data and publication of outcomes among children affected by these programs are essential. Second, studies to quantify the public health burden of SCD need to be conducted. Such studies require interdisciplinary collaboration among different types of researchers and cooperation among different public health programs conducting representative population surveys, including malaria, nutrition, and HIV/AIDS.

In our view, the most efficient and feasible approach to studying the effect of SCD on child survival in Africa is to use reliable, inexpensive laboratory methods with blood specimens routinely collected in large national surveys, such as national nutrition or AIDS indicator surveys. It is important that different regions and both rural and urban populations be represented. The frequency of survival among African children born with SCD appears to be highly variable by time and place, consistent with observations made by Jacob^34^ more than 50 years ago. The probability of early death among children born with SS in sub-Saharan Africa might be as high as 90% in rural areas where access to health care is limited, but closer to 50% in populations with better access to health care and lower exposure to infectious diseases.

We suggest that greater attention to reducing mortality from SCD could help some African governments to achieve their targets with regard to Millennium Development Goal (MDG) number 4: to reduce their under-5 mortality rates by two thirds during 1990–2015.^58^ A recent progress report noted that although under-5 mortality in sub-Saharan Africa has declined by 28% since 1990, the rate of improvement is insufficient to meet the MDG4 target. SCD might be responsible for 5%–16% of under-5 mortality in some areas of sub-Saharan Africa according to published estimates.^59^ Areas of high frequency of the β^S^ allele, shown in dark shading in Figure 1, indicate where homozygous SCD is a major public health problem. As basic public health measures—including improved nutrition and interventions against malaria, pneumonia, and diarrhea—reduce the burden of infectious diseases, it is likely that the absolute burden of mortality attributable to SCD will decrease, but that the relative burden as a fraction of all under-5 mortality actually might rise.^5^

## Figures and Tables

**Table 1:** Predicted SS and β^S^ allele frequency, based on various observed Hb AS genotype frequencies, assuming HWE

AS frequency (%, obs.)	SS frequency (%, HWE)	β^S^ frequency (HWE)
5.0	0.07	0.026
10.0	0.28	0.053
15.0	0.67	0.082
20.0	1.27	0.113
25.0	2.14	0.146
30.0	3.38	0.184
35.0	5.11	0.226
40.0	7.64	0.276

AS, sickle cell trait; β^S^, β-globin S; Hb, hemoglobin; HWE, Hardy-Weinberg equilibrium; obs., observed; SS, sickle cell anemia

**Table 2:** Frequency of Hb SS by age group: selected cross-sectional studies

Study	Study location	Study years	Age groups	N	SS (%)	Comments
Barclay (1971)^46^	Zambia (mining town)	1969–1971	0–11 months	2845	1.3	60% excess mortality by age 12 years
			1–3 years	2200	0.9	
			3–12 years	2306	0.5	
Fleming et al. (1979)^10^	Nigeria (rural Garki)	1970–1972	Newborns	534	2.1	92% excess mortality past age 5 years
			1–4 years	259	0.4	
			5–14 years	637	0.2	
McAuley et al. (2010),^25^ Williams et al. (2009)^41^	Kenya (Kilifi)	1998–2008	0–11 months	782	1.0	90% excess mortality by age 13 years
			12–23 months	282	0.35	
			3–5 years	415	0.24	
			6–13 years	3677	0.09	
Danquah et al. (2010)^47^	Ghana (Northern Region)	2002	0–4 years	1266	0.39	70% excess mortality past age 5 years
			5–10 years	842	0.12	
Simpore et al. (2002)^40^	Burkina Faso	1997–1999	Newborns	HWE	0.25	Data consistent with 50% excess mortality
			Median 9 years	9201	0.13	
Desai et al. (2005)^48^	Kenya (rural Bondo)	1998–1999	Newborns	HWE	1.6	Data consistent with 62% excess mortality prior to the surveys
			0–3 years	2774	0.6	
Allen (1992)^49^	The Gambia (rural)	1988	Newborns	HWE	1.2	Data consistent with 75% excess mortality
			3–8 years	389	0.3	
Cox et al. (2008)^50^	The Gambia (rural)	2003	Newborns	HWE	0.8	Data consistent with 67% excess mortality
			10–72 months	536	0.3	
Sarr et al. (2006)^51^	Senegal (rural Niakhar)	2002–2003	Newborns	HWE	0.5	
			2–10 years	432	None	
Saurin (1984)^52^	Senegal (rural Kégoudou)	1970–1978	Newborns	HWE	1.0	At least 70% excess mortality, but no information by what age
			All ages	596	0.3	

Hb SS, sickle cell anemia; HWE, Hardy-Weinberg equilibrium

## References

[1] Rees D.C., Williams T.N., Gladwin M.T. (2010). Sickle-cell disease. Lancet.

[2] Serjeant G.R. (2005). Mortality from sickle cell disease in Africa. BMJ.

[3] Makani J., Williams T.N., Marsh K. (2007). Sickle cell disease in Africa: burden and research priorities. Ann Trop Med Parasitol.

[4] Diallo D.A. (2008). Sickle cell disease in Africa: current situation and strategies for improving the quality and duration of survival [in French]. Bull Acad Natl Med.

[5] Weatherall D.J. (2010). The inherited diseases of hemoglobin are an emerging global health burden. Blood.

[6] Weatherall D.J., Clegg J.B. (2001). Inherited haemoglobin disorders: an increasing global health problem. Bull World Health Organ.

[7] Obaro S. (2009). Pneumococcal infections and sickle cell disease in Africa: does absence of evidence imply evidence of absence?. Arch Dis Child.

[8] Makani J., Cox S.E., Soka D. (2011). Mortality in sickle cell anemia in Africa: a prospective cohort study in Tanzania. PLoS ONE.

[9] Williams T.N., Obaro S.K. (2011). Sickle cell disease and malaria morbidity: a tale with two tails. Trends Parasitol.

[10] Fleming A.F., Storey J., Molineaux L., Iroko E.A., Attai E.D. (1979). Abnormal haemoglobins in the Sudan savanna of Nigeria. I. Prevalence of haemoglobins and relationships between sickle cell trait, malaria and survival. Ann Trop Med Parasitol.

[11] Molineaux L., Fleming A.F., Cornille-Brogger R., Kagan I., Storey J. (1979). Abnormal haemoglobins in the Sudan savanna of Nigeria. III. Malaria, immunoglobulins and antimalarial antibodies in sickle cell disease. Ann Trop Med Parasitol.

[12] Fleming A.F. (1989). The presentation, management and prevention of crisis in sickle cell disease in Africa. Blood Rev.

[13] Yanni E., Grosse S.D., Yang Q., Olney R.S. (2009). Trends in pediatric sickle cell disease-related mortality in the United States, 1983–2002. J Pediatr.

[14] Quinn C.T., Rogers Z.R., McCavit T.L., Buchanan G.R. (2010). Improved survival of children and adolescents with sickle cell disease. Blood.

[15] Powars D., Overturf G., Weiss J., Lee S., Chan L. (1981). Pneumococcal septicemia in children with sickle cell anemia. Changing trend of survival. JAMA.

[16] Vichinsky E., Hurst D., Earles A., Kleman K., Lubin B. (1988). Newborn screening for sickle cell disease: effect on mortality. Pediatrics.

[17] King L., Fraser R., Forbes M., Grindley M., Ali S., Reid M. (2007). Newborn sickle cell disease screening: the Jamaican experience (1995–2006). J Med Screen.

[18] Rahimy M.C., Gangbo A., Ahouignan G., Alihonou E. (2009). Newborn screening for sickle cell disease in the Republic of Benin. J Clin Pathol.

[19] Grosse S.D., Olney R.S., Baily M.A. (2005). The cost effectiveness of universal versus selective newborn screening for sickle cell disease in the US and the UK: a critique. Appl Health Econ Health Policy.

[20] Grosse S.D., Khoury M., Gwinn M., Higgins J., Ioannidis J., Little J. (2009). Assessing the clinical utility of newborn screening. Human genome epidemiology.

[21] United Nations press office (June 2009). Press conference on raising awareness of sickle-cell anaemia. http://www.un.org/News/briefings/docs/2009/090619_Anaemia.doc.htm.

[22] Modell B., Darlison M. (2008). Global epidemiology of haemoglobin disorders and derived service indicators. Bull World Health Organ.

[23] World Health Organization Regional Office for Africa (22 June 2010). Sickle-cell disease: a strategy for the WHO African Region: Report of the Regional Director.

[24] Piel F.B., Patil A.P., Howes R.E. (2010). Global distribution of the sickle cell gene and geographical confirmation of the malaria hypothesis. Nat Commun.

[25] McAuley C.F., Webb C., Makani J. (2010). High mortality from *Plasmodium falciparum* malaria in children living with sickle cell anemia on the coast of Kenya. Blood.

[26] Tshilolo L., Kafando E., Sawadogo M. (2008). Neonatal screening and clinical care programmes for sickle cell disorders in sub-Saharan Africa: lessons from pilot studies. Public Health.

[27] Christianson A.L., Howson C.P., Modell B. (2006). March of Dimes global report on birth defects: the hidden toll of dying and disabled children.

[28] Ebrahim S.H., Khoja T.A., Elachola H., Atrash H.K., Memish Z., Johnson A. (2010). Children who come and go: the state of sickle cell disease in resource-poor countries. Am J Prev Med.

[29] Edington G.M., Lehmann H. (1954). A case of sickle cell haemoglobin C disease and a survey of haemoglobin C incidence in West Africa. Trans R Soc Trop Med Hyg.

[30] Vandepitte J. (1954). Quantitative and genetic aspects of sickle cell anemia in Leopoldsville [in French]. Ann Soc Belg Med Trop.

[31] Lambotte-Legrand J., Lambotte-Legrand C. (1955). Prognosis of sickle cell anemia in Belgian Congo; based on 300 cases and 150 deaths [in French]. Ann Soc Belg Med Trop (.

[32] Lehmann H., Raper A.B. (1956). Maintenance of high sickling rate in an African community. Br Med J.

[33] Barclay G.P., Huntsman R.G., Robb A. (1970). Population screening of young children for sickle cell anaemia in Zambia. Trans R Soc Trop Med Hyg.

[34] Jacob G.F. (1957). A study of the survival rate of cases of sickle-cell anaemia. Br Med J.

[35] Bernstein S.C., Bowman J.E., Kaptue Noche L. (1980). Population studies in Cameroon: hemoglobin S, glucose-6-phosphate dehydrogenase deficiency and falciparum malaria. Hum Hered.

[36] Akinyanju O.O. (1989). A profile of sickle cell disease in Nigeria. Ann N Y Acad Sci.

[37] Aluoch J.R., Aluoch L.H. (1993). Survey of sickle disease in Kenya. Trop Geogr Med.

[38] Allison A.C. (1956). The sickle-cell and haemoglobin C genes in some African populations. Ann Hum Genet.

[39] Labie D., Richin C., Pagnier J., Gentilini M., Nagel R.L. (1984). Hemoglobins S and C in Upper Volta. Hum Genet.

[40] Simpore J., Pignatelli S., Barlati S., Musumeci S. (2002). Modification in the frequency of Hb C and Hb S in Burkina Faso: an influence of migratory fluxes and improvement of patient health care. Hemoglobin.

[41] Williams T.N., Uyoga S., Macharia A. (2009). Bacteraemia in Kenyan children with sickle-cell anaemia: a retrospective cohort and case-control study. Lancet.

[42] Williams T.N., Mwangi T.W., Wambua S. (2005). Sickle cell trait and the risk of *Plasmodium falciparum* malaria and other childhood diseases. J Infect Dis.

[43] Aidoo M., Terlouw D.J., Kolczak M.S. (2002). Protective effects of the sickle cell gene against malaria morbidity and mortality. Lancet.

[44] Kreuels B., Kreuzberg C., Kobbe R. (2010). Differing effects of HbS and HbC traits on uncomplicated falciparum malaria, anemia, and child growth. Blood.

[45] Masmas T.N., Garly M.L., Lisse I.M. (2006). Inherited hemoglobin disorders in Guinea-Bissau, West Africa: a population study. Hemoglobin.

[46] Barclay G.P. (1971). Sickle cell anaemia in Zambia. Trans R Soc Trop Med Hyg.

[47] Danquah I., Ziniel P., Eggelte T.A., Ehrhardt S., Mockenhaupt F.P. (2010). Influence of haemoglobins S and C on predominantly asymptomatic *Plasmodium* infections in northern Ghana. Trans R Soc Trop Med Hyg.

[48] Desai M.R., Terlouw D.J., Kwena A.M. (2005). Factors associated with hemoglobin concentrations in pre-school children in western Kenya: cross-sectional studies. Am J Trop Med Hyg.

[49] Allen S.J., Bennett S., Riley E.M. (1992). Morbidity from malaria and immune responses to defined Plasmodium falciparum antigens in children with sickle cell trait in The Gambia. Trans R Soc Trop Med Hyg.

[50] Cox S.E., Doherty C.P., Atkinson S.H. (2008). Haptoglobin genotype, anaemia and malaria in Gambian children. Trop Med Int Health.

[51] Sarr J.B., Pelleau S., Toly C. (2006). Impact of red blood cell polymorphisms on the antibody response to *Plasmodium falciparum* in Senegal. Microbes Infect.

[52] Saurin W. (1984). Natural selection study in small size populations. The drepanocytosis case in Madinka and Bedik from the Kedougou region (Eastern Senegal) [in French]. Bull Mém Soc d'Anthrop Paris.

[53] Athale U.H., Chintu C. (1994). Clinical analysis of mortality in hospitalized Zambian children with sickle cell anaemia. East Afr Med J.

[54] Tshilolo L., Aissi L.M., Lukusa D. (2009). Neonatal screening for sickle cell anaemia in the Democratic Republic of the Congo: experience from a pioneer project on 31 204 newborns. J Clin Pathol.

[55] Munyanganizi R., Cotton F., Vertongen F., Gulbis B. (2006). Red blood cell disorders in Rwandese neonates: screening for sickle cell disease and glucose-6-phosphate dehydrogenase deficiency. J Med Screen.

[56] Mutesa L., Boemer F., Ngendahayo L. (2007). Neonatal screening for sickle cell disease in Central Africa: a study of 1825 newborns with a new enzyme-linked immunosorbent assay test. J Med Screen.

[57] Ohene-Frempong K., Oduro J., Tetteh H., Nkrumah F. (2008). Screening newborns for sickle cell disease in Ghana. Pediatrics.

[58] United Nations (2000). United Nations Millennium Declaration, resolution 55/2.

[59] World Health Organization (17–19 May 2006). Report of a joint WHO–March of Dimes meeting: management of birth defects and haemoglobin disorders.

